# What Role Does Geragogy Play in the Delivery of Digital Skills Programs for Middle and Older Age Adults? A Systematic Narrative Review

**DOI:** 10.1177/07334648221091236

**Published:** 2022-05-11

**Authors:** Jessica R. Gates, Gemma Wilson-Menzfeld

**Affiliations:** 1 Faculty of Health and Life Sciences, Northumbria University, Newcastle upon Tyne, UK

**Keywords:** technology, aging, geragogy, gerontechnology, digital literacy, skills training, digital divide

## Abstract

This systematic narrative review aimed to explore the implementation and delivery of digital skills programs for middle and older age adults; and understand the presence of adult learning theory (namely, geragogy/critical geragogy) in their delivery. A database search was undertaken to examine international literature, published between 2010 and 2020. From 1,713 papers identified during the database searches, 17 papers were included. Thematic synthesis was used to analyze the papers in this review. Themes were generated relating to the implementation and delivery of digital skills programs: negative perceptions of aging; the learning environment; and value of technology. The role of geragogy/critical geragogy is not explicit in the delivery of digital skills programs in this review but has an underlying thread of empowerment and embodies the ethos of these learning theories to some extent. The findings of this review have been used to develop recommendations for delivering digital skills to older adults.

## Background

The “digital divide” is defined as the gap between those who are digitally included and those who are digitally excluded. Individuals are more likely to be digitally excluded if they have lower educational attainment (Bergström, 2017; Cresci & Jarosz, 2010; Hargittai & Dobransky, 2017; Neves et al., 2013a), lower health status (Age UK, 2015, 2018; Cresci & Jarosz, 2010; Matthews et al., 2019), and are disabled (Choi & Dinitto, 2013). Older adults are also more likely to be digitally excluded than younger adults (Bergström, 2017; Choi & Dinitto, 2013; Friemel, 2016; Gilleard & Higgs, 2008; Gordon & Hornbrook, 2018; Hargittai & Dobransky, 2017; Matthews et al., 2019; Yoon et al., 2020), and whilst digital exclusion is not solely an issue related to advancing age, there are various factors which heighten the risk of digital exclusion for older adults including the cross-sectional inequalities described above (Age UK, 2018).

Digital exclusion is categorized across three levels: access, skills and usage, and the tangible outcomes from internet use which can result in offline benefits, specifically, economic, social, political, institutional, and educational (Blank & Groselj, 2014; Scheerder et al., 2017; *A*. van Deursen & E. j. Helsper, 2015). These three factors have the potential to prevent individuals fully participating in society (Schejter et al., 2015) through lack of access to services such as internet banking, digital NHS services, or benefit applications (Age UK, 2018).

In terms of lack of access, it is important to not only consider both physical access to the digital technology itself (e.g., a smartphone or tablet), but also material access by having the ability to maintain digital access such as up-to-date hardware, software, and internet connectivity (A. J. van Deursen & van Dijk, 2019). This lack of access may be due to cost, lack of interest, lack of perceived need, privacy concerns, or lack of skills to use it (Baker et al., 2020). A. van Deursen and van Dijk (2008) classified digital skills into four categories: Operational skills, that is, the skills required to use digital technologies; Formal skills, that is, the skills required to manage the structures of digital media; Information skills, that is, the skills required to find information online; and Strategic skills, that is, the skills to translate digital information to personal and professional development. These skills are broader than basic computer skills and account for the ability to grasp online content (A. J. Van Deursen et al., 2014) arguably reflecting wider models of learning theory and pedagogy.

Lifelong learning is a means to empower individuals, in developing new skills and reaching personal fulfillment (Withnall, 2009) including developing digital skills which may lead to reduced digital exclusion. However, models of learning in later life often focus on the psychological deficit model (Formosa, 2012) and there is much debate as to whether older adults (over 50 years old) are marginalized from wider theoretical frameworks concerned with lifelong learning (Findsen & Formosa, 2012; Formosa, 2012).

Pedagogy differs to geragogy and critical geragogy with the latter concepts considering a more nuanced learning theory that targets older adults’ learning and acknowledges older adults’ distinct physical, emotional, and social learning needs (Formosa, 2002, 2011; Lebel, 1978; Wright & Wright, 2016). Critical geragogy demands consideration of transformative conditions that promote disempowerment, and “for unsettling learners’ assumptions that they cannot effect social change” considering the importance of self-directed and self-regulated learning as opposed to the power of learning being held by others (teachers).

It is imperative to consider learning theory, particularly geragogy and critical geragogy, in digital skills training for older adults, as these learning theories promote empowerment and autonomy, as well as to promoting peer-learning and personalised learning (Formosa, 2012; Glendenning & Battersby, 1990). This is particularly important when engaging groups of older adults most at risk of digital exclusion. Importantly, older adults are a heterogenous group, and therefore differ considerably in their digital access, technology adoption, and digital skills (Niehaves & Plattfaut, 2014; *A.*van Deursen & E. j. Helsper, 2015). This heterogeneity also concerns learning, and the learning environment in which these digital skills are acquired. Geragogy has been critiqued by those advocating for critical geragogy as being a top-down approach which corroborates with the notion of older adults being one homogenous group (Findsen & Formosa, 2012) as opposed to celebrating the diversity of individuals in later life (Creech & Hallam, 2015).

Whilst it is clear that digital learning in later life is central to reducing digital exclusion, there is currently no review which synthesizes the evidence in this area or considers the role of learning theory in older adults’ digital learning. Evidence synthesis is imperative when developing policy and practice recommendations, to bring together current practice, and to develop guidance which will improve practice and increase consistency across programs. This review therefore sought to explore existing evidence of digital support programs for older adults. This review had two main aims: 1) To explore the implementation and delivery of digital skills support programs for middle and older age adults and 2) To understand the presence of adult learning theory (namely, geragogy/critical geragogy) in the delivery of digital skills support programs for middle and older age adults.

## Method

In order to allow for the inclusion of evidence from multiple sources, a systematic narrative review design was chosen (Snilstveit et al., 2012). Search terms were constructed using the PICO mnemonic (patient/population, intervention, control, outcome; Table 1). Suitable databases were identified and selected for a comprehensive search (Table 2). Regarding the patient or population element, we included both the terms “middle age” and “older adult,” among other variations on the terms, due to the conflicting arguments as to what age range constitutes an older adult.

**Table 1.** The PICO Framework to Develop a Search Strategy Used for the Systematic Research.

P	Patient or Population	“Middle Age” OR “Older Adult” OR “Generation x” OR “Baby Boomers” OR “Elder*” OR “Senior” OR “Pensioner” OR “Age*” OR “Silver Surfer”
I	Intervention	“Education*” OR “programme” OR “group” OR “help” OR “support” OR “digital literacy” OR “learning technology” OR “educational intervention” OR “teaching” OR “training”
C	Comparison (if applicable)	Not applicable
O	Outcome	“Internet use” OR “technology” OR “computer*” OR “online” OR “tablet” OR “smartphone” OR “digital” OR “social media” OR “gerontechnology”

**Table 2.** Search Strategy for Systematic Search.

Source	Web of Science ASSIA Science Direct Grey Literature	CINAHL PsychARTICLES SCOPUS Reference List Searching
Search Field	Title, Abstract, Keywords	
Language	English only	
Inclusion	International literature Qual/quant/mixed methods Implementation research Evaluation research	
Exclusion	Not English language Other systematic reviews Conference abstracts	
Year of publication	2010 onwards	

**Figure 1.**
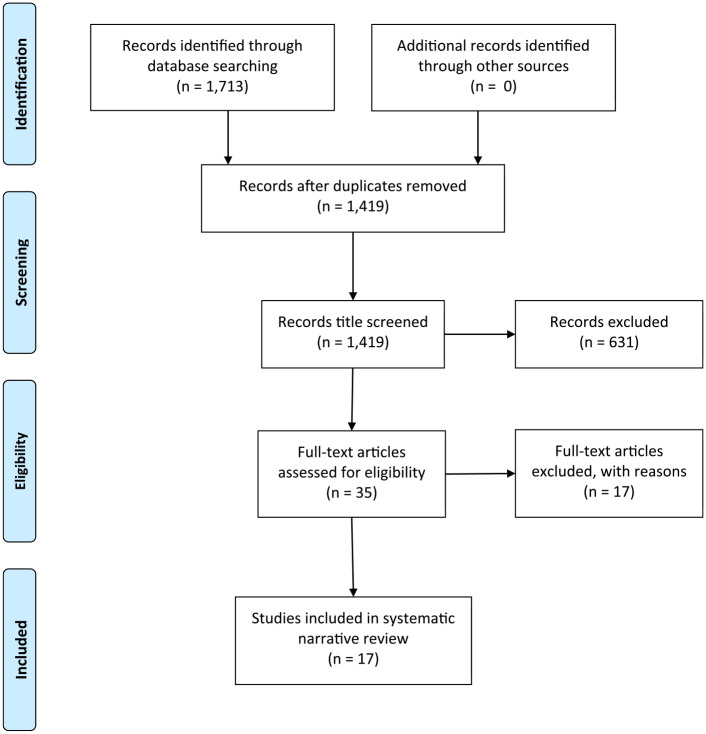
PRISMA diagram of papers identified during search process.

A total of 1,713 papers were identified through the database searching exercise, duplicates were removed, and suitable articles were screened (Figure 1).

The Critical Appraisal Skills Programme tool was used to examine the quality of the included papers (https://casp-uk.net/casp-tools-checklists/). A total of 17 papers were included in the review. Each paper was then analyzed using thematic synthesis (Thomas & Harden, 2008). Utilising the three-stage method proposed by Thomas and Harden (2008), the authors carried out line-by-line coding of the findings section of each study, extrapolating findings related to the current review’s aim. One author reviewed each paper, however, five papers were reviewed by both authors and checked for quality in coding. From these codes, descriptive themes were then generated, before being further developed into analytical themes and sub-themes. The analytical themes and sub-themes are presented below.

## Results

17 papers were included for review (Supplementary Table 3). Of these papers, five were qualitative (Sajay Arthanat, 2019; Chiu et al., 2019; LoBuono et al., 2019, 2020; Tomczyk et al., 2020), three were quantitative (Czaja et al., 2012; Loi et al., 2017; Xie, 2012), seven papers were mixed methods (Beh et al., 2018; Castro Rojas et al., 2018; Fields et al., 2020; Gould et al., 2020; S. N.; Leedahl et al., 2019a; Seguí et al., 2019; Seo et al., 2019), and two papers were essays (Brown & Strommen, 2018; Jobling, 2014).

Three themes were generated across the papers: Negative perceptions of aging; the learning environment; and value of technology.

### Negative Perceptions of Aging

Perceptions of aging had an impact upon the delivery of the programs, both from the perspective of users and deliverers.

In terms of the service approach, seven papers utilized intergenerational learning (S. Arthanat, 2019; Brown & Strommen, 2018; Chiu et al., 2019; Skye N. Leedahl et al., 2019; LoBuono et al., 2019, 2020; Seguí et al., 2019), one paper used a peer tutor approach (Jobling, 2014), and three papers used professional tutors (Loi et al., 2017; Seo et al., 2019; Xie, 2012). Additionally, seven of these papers used a one-to-one teaching style (Arthanat et al., 2019; Brown & Strommen, 2018; Fields et al., 2020; S. N.; Leedahl et al., 2019b; LoBuono et al., 2020, 2019; Seguí et al., 2019), while four of these papers conducted group activities (Beh et al., 2018; Chiu et al., 2019; Czaja et al., 2012; Xie, 2012).

The intergenerational learning environment was beneficial for both the younger and older participants and encouraged bidirectional learning (Seguí et al., 2019). The intergenerational approach allowed for negative conceptualizations of aging to be challenged, which in turn helped improve the facilitators’ approach to training older adults (Brown & Strommen, 2018; Seguí et al., 2019). The older participants found enjoyment in interacting with younger tutors (S. N. Leedahl et al., 2019a).

Five papers cited age-specific barriers to digital learning (Arthanat et al., 2019; Brown & Strommen, 2018; Gould et al., 2020; LoBuono et al., 2019; Tomczyk et al., 2020). The notion of subjective aging arose and how this may predict learning outcomes (Brown & Strommen, 2018). Consideration was given to enhancing the learning of older adults, by providing information on aging, disability, learning styles, and strategies (Brown & Strommen, 2018). Furthermore, it has been suggested that successful learning can be fortified by understanding and adapting to the preferences, cognitive, and physical needs of the older adults (LoBuono et al., 2019). Negative attitudes related to the learners’ age, can also have an impact on learning and can potentially lead to self-exclusion from the digital space (Tomczyk et al., 2020).

### The Learning Environment

The learning environment was an important theme across these papers, with reflections on notions of lifelong learning and the importance of the instructor.

Twelve papers discussed the importance of the learning environment (Arthanat et al., 2019; Castro Rojas et al., 2018; Chiu et al., 2019; Czaja et al., 2012; Fields et al., 2020; Jobling, 2014; S. N.; Leedahl et al., 2019a; LoBuono et al., 2020; Loi et al., 2017; Seguí et al., 2019; Seo et al., 2019; Xie, 2012). There are a number of practical considerations regarding the learning environment. Structured learning environments with well-defined activities are preferred (Castro Rojas et al., 2018), whilst remaining flexible and having the ability to incorporate different activities (S. N. Leedahl et al., 2019a). Ensuring there is sufficient time in the session to practice one skill was beneficial, compared with being overwhelmed by too much information (Jobling, 2014). Continued engagement rather than one-off sessions also facilitated learning (Loi et al., 2017). Class size, being able to practice with the tools and being given handouts to take home, all assisted in the learning process (Xie, 2012).

Thirteen papers highlighted the importance of the instructor (Arthanat et al., 2019; Brown & Strommen, 2018; Castro Rojas et al., 2018; Chiu et al., 2019; Fields et al., 2020; Gould et al., 2020; Jobling, 2014; S. N.; Leedahl et al., 2019a; LoBuono et al., 2019; Loi et al., 2017; Seo et al., 2019; Tomczyk et al., 2020; Xie, 2012). Program success was dependent upon volunteer engagement and the training provided for volunteers (Brown & Strommen, 2018). Instructors should generate a respectful, friendly and safe environment and ensure learners are comfortable with asking questions (Castro Rojas et al., 2018). Important skills for instructors include patience, empathy, and positivity (Jobling, 2014). An interest to connect personally with the learners (Jobling, 2014) and developing trust and a good rapport with the learners was vital to helping them overcome barriers (Arthanat et al., 2019). The data suggests the success of an educational program can hinge upon the instructor’s teaching style and personal traits (Seo et al., 2019).

Four papers referenced lifelong learning (LoBuono et al., 2019; Seo et al., 2019; Tomczyk et al., 2020; Xie, 2012). Increasing digital literacy and technology-based skills was associated with promoting productive aging (LoBuono et al., 2019). Developing these skills allows older adults to pursue their own interests in later life, such as using technology for writing books, broadening their education, or applying for jobs (LoBuono et al., 2019). Additionally, improving digital literacy also allows older adults to engage with health care information, which is increasingly important as technology is further integrated into health services (Xie, 2012).

### Value of Technology

The value that older adults place on technology and learning new technology-based skills was crucial, with both users and deliverers noting the benefits of understanding their motivations to use technology, tailoring and personalizing program sessions to the individual, and the benefit of integrating programs into other services.

Seven papers referred to motivation to use technology (Arthanat et al., 2019; Castro Rojas et al., 2018; Czaja et al., 2012; Fields et al., 2020; Loi et al., 2017; Seo et al., 2019; Tomczyk et al., 2020). Motivation to learn new technologies are often interlinked with the perception of usefulness and relevance (Castro Rojas et al., 2018). Concerns around online security and privacy hinder motivation to engage with learning new technologies, and these concerns should be addressed in learning programs (Seo et al., 2019). Social connectivity was also a motivation to use technology and may be a gateway to technology adoption (Arthanat et al., 2019). Other motivations included perceived necessity, learning something new, engaging with their interests, accessing health/government information, and reducing feelings of loneliness (Czaja et al., 2012; Fields et al., 2020).

Ten papers noted the benefits of personalization, in that personalizing learning programs allowed learners to see the value of digital technologies (Arthanat et al., 2019; Beh et al., 2018; Brown & Strommen, 2018; Castro Rojas et al., 2018; S. N.; Leedahl et al., 2019b; LoBuono et al., 2020, 2019; Seguí et al., 2019; Seo et al., 2019; Xie, 2012). Individualized lessons allowed learners to develop skills most relevant to them and on devices they already owned, making it easier for instructors to problem-solve (Brown & Strommen, 2018). Engagement was facilitated by perceived usefulness and how meaningful the activity was to the individual (Arthanat et al., 2019). Incorporating pre-existing interests into the curriculum also facilitated learning (Beh et al., 2018). Personalization also allowed for the individual’s unique learning style and preferences to be taken into account (Seo et al., 2019).

Five papers cited integration into other services (Brown & Strommen, 2018; Fields et al., 2020; Seo et al., 2019; Tomczyk et al., 2020; Xie, 2012). Collaborating with partner sites was a beneficial approach to delivering learning to older adults (Brown & Strommen, 2018). Some partner sites, such as community centres/non-profits, have existing technology training programs and may be willing to pilot new approaches (Brown & Strommen, 2018). Academic-community partnerships are also beneficial, particularly for those organisations lacking resources (Seo et al., 2019). Integration into other services can also enhance participation (Xie, 2012) and can be especially successful when working in vulnerable populations (Fields et al., 2020).

## Discussion

This study aimed to explore the implementation and delivery of digital skills support programs for older adults; as well as understand the presence of adult learning theory (namely, geragogy) in the delivery of digital skills support programs for older adults. The 17 papers included in this review highlighted the importance of negative perceptions of aging, the learning environment, and the value of technology.

Internalized negative perceptions and stereotypes of aging were a major barrier throughout the presented evidence (Arthanat et al., 2019; Brown & Strommen, 2018; Gould et al., 2020; LoBuono et al., 2019; Tomczyk et al., 2020), and it is clear that this must be addressed through the delivery of digital support programs. Aging preconceptions is a notion considered in critical geragogy, and is one of the barriers with implementing this theory into practice (Formosa, 2012). Previous research suggests that some older adults make negative assumptions about their abilities simply because of their older age (Good Things Foundation and Talk, 2018; (Neves et al., 2013b) and this is heightened through cross-sectional inequalities, which itself heightens the risk of digital exclusion (Age UK, 2018). Furthermore, these views are often worsened by prior negative experiences of learning new digital skills (Centre for Ageing Better, 2018). This stresses the importance of the learning environment, and the delivery of digital skills training, as well as recognizing individual factors that contribute to an individual’s learning. Embracing the ethos of critical geragogy, and in particular the notion of empowerment, would serve to challenge and dismantle these internalized negative perceptions of aging and improve the learning environment.

Studies in this review suggest older adults thrive when there is positive rapport between teacher and learner (Raymond J Wlodkowski, 1999). Formosa (2018) suggests that peer teaching is generally considered the most effective method in later-life learning. Simson et al. (2002) found that the peer teaching experience is a positive one, with teachers noting personal satisfaction and intellectual stimulation (Simson et al., 2002). Intergenerational learning sits against the ethos of critical geragogy, which stresses the importance of peer-learning and empowerment (Formosa, 2012); however, findings from this review exhibit positive experiences of intergenerational learning (Brown & Strommen, 2018; S. N. Leedahl et al., 2019a; Seguí et al., 2019). Findings from this review also demonstrated positive outcomes of intergenerational learning for the facilitator as the intergenerational learning environment encouraged bidirectional learning for both older participants and younger facilitators (Seguí et al., 2019). Whilst younger facilitators might have held beliefs of the participants’ abilities before the class, these were challenged throughout the programs. While we understand the importance of peer-learning within critical geragogy, we argue there is also space for intergenerational learning approaches within this realm. It can be an opportunity to actively challenge stereotypes held by both the facilitators and learners.

Digital skills programs have the potential to genuinely empower older adults (Ferreira et al., 2016) and it is therefore important that these programs seek to build confidence and increase feelings of self-efficacy among learners, building this into the program from the outset. Research suggests low self-efficacy can limit technology use which can be problematic for learners (Wilson, Gates, Vijaykumar, & Morgan, 2021; Age UK, 2015; Centre for Ageing Better, 2018). This review found the learning environment to be particularly influential in gaining digital skills and building confidence. Facilitating a smooth transition back into the classroom, after what may have been an extended absence, can help ensure the learning environment feels like an empowering and welcoming space (Baringer et al., 2004). It would also be prudent to consider the social aspect of these digital skills programs as providing learners the opportunity to meet new people through these classes may increase engagement with the class and increase confidence (Good Things Foundation and Talk Talk, 2018; (Zaidman & Tinker, 2016). Therefore, teaching older adults provides an opportunity to collaborate with the learners, and create a sense of social inclusion (R.J Wlodkowski, 2008).

Older adults are a diverse cohort, yet they are often viewed as one homogenous group with the same needs. Viewing older adults as one homogenous group is problematic for a number of reasons, but when it comes to learning this can be a significant barrier. It is critical that digital skills training considers critical geragogy to empower older learners, particularly those at most risk of digital exclusion. Critical geragogy stresses the importance of lifelong learning for older adults, and recognises that older adults inhabit different physical, emotional and social realms compared with other age groups (Formosa, 2018). Some approaches assume that any type of learning improves the quality of life for the older adult (Formosa, 2012), but as discussed previously this is not one homogenous group. In contrast, critical geragogy celebrates the diversity among older adults and suggests later life can be a time of creativity (Hickson & Housley, 1997) and Formosa (2002) argues that condescending practices should be rejected, and replaced with dialog, negotiation, reflection and promoting ownership over the learning experience (Formosa, 2002).

One of the crucial aspects of this review found that personalization of programs not only supported the learning environment in that it allowed for the individual’s unique learning style and preferences to be taken into account (Seo et al., 2019), but also the value of the program. This strengthens A. J. Van Deursen and E. J. Helsper (2015) theory of the digital divide, in demonstrating the importance of recognizing the tangible benefits of being online. To engage learners, and older learners, the materials must reflect the “real world” rather than being abstract and impractical (Peterson, 1983). More recent evidence in the digital skills arena strengthens the notion in that non-personalized and traditional ICT courses are less effective in getting people online (Age UK, 2018). This review demonstrated that personalizing learning programs enabled learners to see the value of digital technologies (Arthanat et al., 2019; Beh et al., 2018; Castro Rojas et al., 2018; S. N.; Leedahl et al., 2019a; LoBuono et al., 2020, 2019; Seguí et al., 2019; Seo et al., 2019; Xie, 2012), and allowed them to choose devices they already owned, and to learn skills most useful to them (Brown & Strommen, 2018).

Understanding the value of the technology is imperative in reducing digital exclusion in two ways. First, demonstrating the value of technology to individuals is important in order to increase knowledge and understanding of the tangible outcomes from internet use and ultimately increasing the want to participate, (Blank & Groselj, 2014; Scheerder et al., 2017; *A.* van Deursen & E. j. Helsper, 2015). Value sometimes may only be recognized once the individual has started using the device (Tsai et al., 2015) and therefore may reduce the motivation to use technology. Furthermore, the evidence from this review and beyond highlights the importance of considering individual motivations, perceived value, potential impact, and need (Arthanat et al., 2019; Centre for Ageing Better, 2018; Castro Rojas et al., 2018; Centre for Ageing; Czaja et al., 2012; Good Things Foundation and Talk Talk, 2018; Seo et al., 2019). Value and personalization of the learning offer are therefore closely intertwined.

In support of our findings, previous research has proposed a number of principles for engaging older adults in technology use: flexibility, relevance, the right pace, repetition, reflection, the right language, one-to-one support, time to build relationships, ongoing support, and co-design (Age UK, 2018; Centre for Ageing Better, 2018). In addition to these principles for engaging older adults with technology use, it is important to also consider the wider components to designing and delivering a digital skills program. The evidence in this review suggests that there are many benefits to exploring existing services and infrastructures. Collaborating with other organisations can help facilitate a co-creation or participatory approach to the development of these programs, with all partners benefitting from shared knowledge and resources.

### Implications

When considering delivering a digital skills program to older adults, there is a clear intersection with negative perceptions of aging, the learning environment, and the value of technology. Taken together, we argue that addressing these components can improve not only the procedures, but also the experiences of those delivering the program, those receiving the program and help improve sustainability of programs over time.

**Figure 2.**
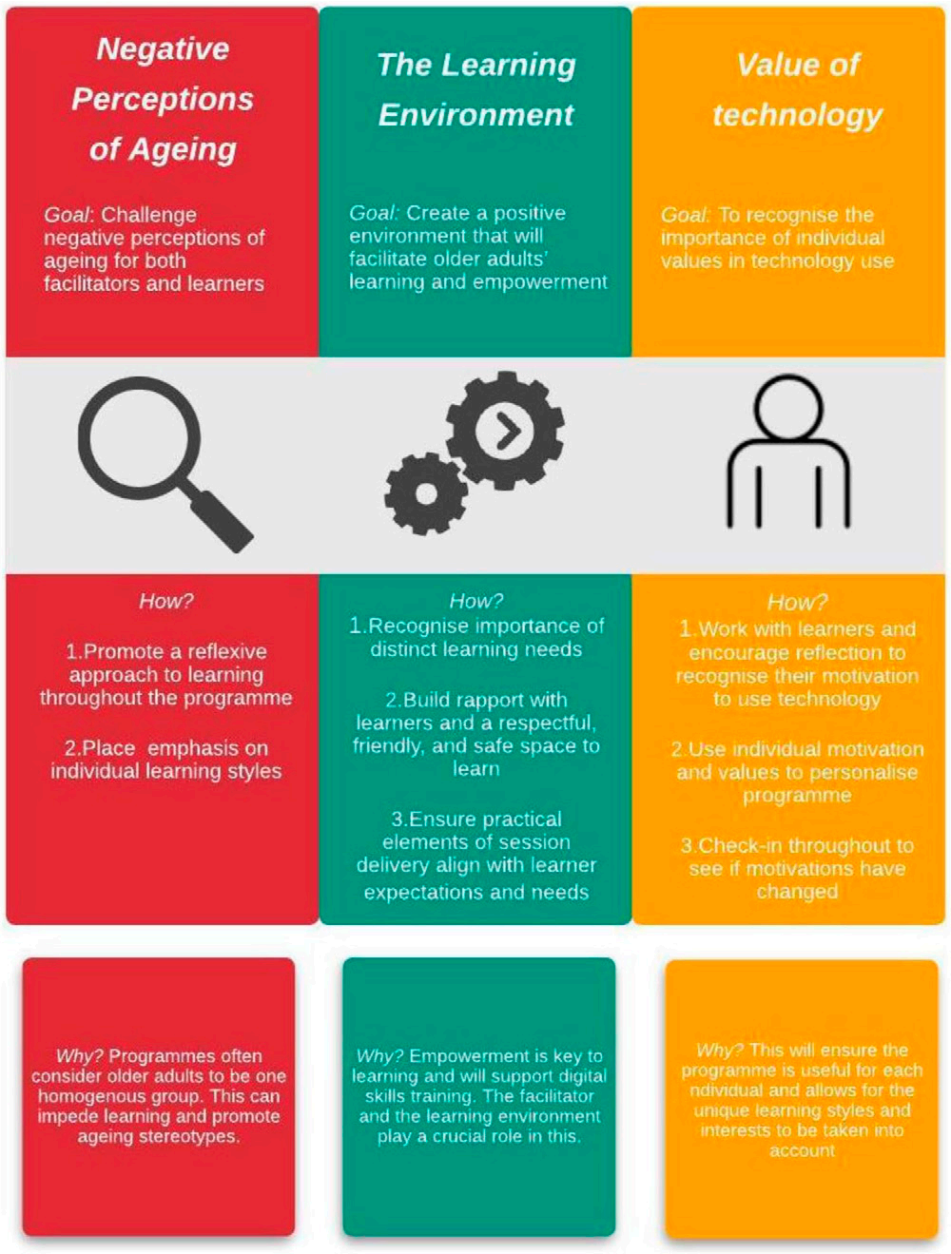
Critical geragogy in Action: Recommendations for delivering digital skills to older adults.

The literature on geragogy and critical geragogy should be considered when developing digital skills support programs for older adults, with an emphasis on providing older adults greater control over their own knowledge. It is evident that this theory has not been overtly translated into practice through the reviewed papers in this article. Reflections should be undertaken throughout, to promote the development of interventions through the lens of critical geragogy to support empowerment, autonomy, peer-learning, and personalized learning. Figure 2 below outlines recommendations for delivering digital skills to older adults, with an emphasis on putting critical geragogy into action.

### Strengths and Limitations of the Review

Many systematic reviews in this area understandably focus on the outcomes of the learners; however, this systematic narrative review provides a unique and necessary perspective of program delivery. This approach has allowed the authors to develop recommendations for those working with older adults to deliver digital services, thus making the review more accessible and practical.

There are some limitations to the review however that need addressing. Many of the included papers covered staff experiences, but this was often in conjunction with the user experience and at times could be viewed as a tokenistic add on. Nevertheless, the literature did include staff experiences and we were able to utilize this research. Future research however should seek to specifically address the experiences of those delivering digital skills programs.

Additionally, research papers were only included with publication dates ranging from 2010 to 2020. Due to rapid technological advancements over the last 10 years, contemporary literature was deemed more salient. However, it is possible that important articles were excluded due to this cut-off date.

While discussing and interpreting the review findings through the lens of geragogy, only one paper (Tomczyk et al., 2020) referenced adult learning theories and the notion of geragogy and critical geragogy. While the authors aim to explore the role that geragogy plays in the delivery of digital skills programs for middle and older age adults, the majority of papers did not acknowledge these theories. We argue this is a limitation of both the literature and the programs themselves, as these key learning theories were not considered when developing learning programs for older adults.

Finally, it is important to consider that this review searched for studies across a 10-year timespan, although only four studies were over 4 years old. Despite the rapid digital advancements over this period, the reason for this inclusion is due to the focus on the learning environment, as opposed to the technology itself. There were no fundamental differences between studies based on time published, showing the relative consistency in approach, despite technological changes.

## Conclusion

This systematic narrative review aimed to examine the role of geragogy in the delivery of digital skills programs for middle and older age adults. If this was examined strictly based on whether the literature acknowledges the theories of geragogy/critical geragogy, the answer would be that geragogy plays no explicit role in the delivery of these programs. However, if you unpick the themes relating to the implementation and delivery of these programs, the themes all lie within the ethos of critical geragogy. The main themes: negative perceptions of aging; the learning environment; and value of technology, all have an underlying thread of empowerment and embody geragogy.

It is essential to consider why delivering digital skills programs effectively is important. The end goal is to narrow the digital divide, giving older adults the ability to engage with digital tools as they see fit. While the end goal is important, so is the process. Ensuring the learning experience is suitable for the learners receiving it should be the most basic of requirements. Removing the misconception that “any type of learning will do” is crucial to improving the services that are available across the globe and embracing geragogy/critical geragogy in the development of services will help achieve this.

## Supplemental Material

sj-docx-1-jag-10.1177_07334648221091236 – Supplemental material for What Role Does Geragogy Play in the Delivery of Digital Skills Programs for Middle and Older Age Adults? A Systematic Narrative Review Click here for additional data file. Supplemental material, sj-docx-1-jag-10.1177_07334648221091236 for What Role Does Geragogy Play in the Delivery of Digital Skills Programs for Middle and Older Age Adults? A Systematic Narrative Review by Jessica R. Gates and Gemma Wilson-Menzfeld in Journal of Applied Gerontology

sj-docx-2-jag-10.1177_07334648221091236 – Supplemental material for What Role Does Geragogy Play in the Delivery of Digital Skills Programs for Middle and Older Age Adults? A Systematic Narrative Review Click here for additional data file. Supplemental material, sj-docx-2-jag-10.1177_07334648221091236 for What Role Does Geragogy Play in the Delivery of Digital Skills Programs for Middle and Older Age Adults? A Systematic Narrative Review by Jessica R. Gates and Gemma Wilson-Menzfeld in Journal of Applied Gerontology
