# Supplementary material for: What Role Does Geragogy Play in the Delivery of Digital Skills Programs for Middle and Older Age Adults? A Systematic Narrative Review
Source: J Appl Gerontol. 2022 May 11;41(8):1971–80. doi: 10.1177/07334648221091236 (PMC9364233; doi:10.1177/07334648221091236)
Supplement: sj-docx-1-jag-10.1177_07334648221091236 – Supplemental material for What Role Does Geragogy Play in the Delivery of Digital Skills Programs for Middle and Older Age Adults? A Systematic Narrative Review [file sj-docx-1-jag-10.1177_07334648221091236.docx]

**Table 3**. Summary and details of studies included.

| **AUTHOR & YEAR** | **AIM** | **DESIGN** | **DATA COLLECTION** | **PROGRAMME** | **SERVICE APPROACH** | **ENVIRONMENT** | **PARTICIPANTS** |
| --- | --- | --- | --- | --- | --- | --- | --- |
| Arthanat, S., Vroman, K.G., Lysack, C., & Grizzetti, J. (2019) | This study aimed to identify and conceptualize barriers and strategies for  effective implementation of information communication technology training for older adults. | Qualitative | Interviews and focus groups | This 3-month iPad training programme involved a group information session. Older adults were then paired with a “personal coach” (occupational therapy graduate).  After this session the programme was delivered 1:1 at home. The coaches were also available by email or phone.  Participants noted what was valuable for them to learn during the training sessions.  The programme is described in: Arthanat, Vroman & Lysack (2016). A homebased individualized information communication technology training program for older adults: a demonstration of effectiveness and value, Disability and Rehabilitation: Assistive Technology, 11, 4, 316-324, DOI: 10.3109/17483107.2014.974219 | 1:1 intergenerational sessions | Home-based with one introductory group session. | 14 community care providers, 35 graduate occupational  therapy students and 12 older adult participants who completed  the pilot individualized home-based ICT training programme |
| Beh, J., Pedell, S., & Mascitelli, B. (2018) | This study aimed to examine older adults’ pre-existing interests and approaches  to support learning and uptake of mobile touch screen  technologies. | Mixed | Interviews; focus groups; pre, mid, post study questionnaires | Four studies were carried out.  Exploratory study – An activity group was carried out over a period of 12 weeks (2-hour weekly visits). This aimed to understand pre-existing interests of using technology.  Comparative study - Four different approaches were compared over a period of 12 weeks, in terms of teaching focusing on differing levels of interest.  Conceptual phase - an environment was required to trial and translate the theory that was developed into practical guidelines and tools.  In-depth study – Classes were run on an ad-hoc basis and were informed by previous study phases. | Group activity | Community setting | 131  older adults (>65 years), five staff members, and eight teachers were involved across the  four studies. |
| Brown & Strommen (2018) | The purpose of this article was to describe the development of an  intergenerational technology training program that utilized a subjective aging intervention to enhance  technology learning among older adults. | Descriptive article, pilot study. | No reportable data. | The TeachSD Toolkit | 1:1, Intergenerational | N/A | Participant numbers not reported.  Adult Learners – aged 50+.  Young trainers – aged 14-24. |
| Castro Rojas et al (2018) | The study aimed to provide context-sensitive design principles that could guide the design of  suitable learning interventions to enable older people to use ICTs for cognitive activity and social  interaction. | Mixed Methods | Data from relevant literature, a focus group with older adults, interviews with professionals teaching older adults. | N/A | N/A | N/A | Focus group (seven participants, ages not reported).  Interviews (two participants, trainers, ages not reported). |
| Chiu, C-J., Tasi, W-C., Yang, W-L., Guo, J-L. (2019) | This study aimed to investigate the practical teaching status at a senior learning centre, as well as course preparation, course design orientation, and the teaching behaviour of the instructors.  This study also investigated the use of teaching strategies and  mapped a real situation in the field at a senior learning center for internet technology. | Qualitative | Participatory observations and interviews. | Instructors of multiple programmes were included in this study. | Group activity; Intergenerational | Community centres | Six instructors between 30-40 years |
| Czaja, S.J., Lee, C.C., Branham, J.m., & Remis, P. (2012) | This study aimed to evaluate a community-based  computer and Internet training programme, to provide recommendations for programme  refinement, and to gather information  for sustainability. | Quantitative | Two-group design. Technology questionnaires; and computer attitude / knowledge / assessment questionnaires. | The programme comprised of 12 2-hour sessions across two courses: Introduction to computers and Introduction to the internet. The course was structured to cover simple to more complex concepts. The course was highly interactive and a manual was provided. | Group activity (maximum of 10 per class) | Community setting | 196 older adults assigned (aged 40-90) assigned to training or wait-list groups. |
| Fields, J., Cemballi, A.G., Michalec, C., Uchida, D., Griffiths, K., Cares, H., Cuellar, J., Chodos, A.H., & Lyles, C.R. (2020) | This study aimed to evaluate the programme’s effect on loneliness, perceived social support, and technology use. This study also aimed to identify implementation and dissemination lessons for future interventions. | Mixed | Two-group design. Surveys and interviews. | Participants received all necessary equipment (iPad, broadband, etc) before beginning the programme. Eight weekly digital training sessions were provided along with a learner book. | 1:1 | Home-based sessions | 57 participants completed post-training surveys. 20 participants carried out baseline interviews, 15 completed post-training interviews. |
| Gould, C.E., Loup, J.R., Scales, A.N., Juang, J., Carlson, C., Ma, F., & Sakai, E.Y. (2020) | This study designed and evaluated patient education materials teaching how to download apps and the basics of  mobile device use. | Mixed | Survey | Based on evidence in the field, how-to-download guides, mobile device information  Guides, and step-by-step app guides were developed and evaluated. | N/a | N/a | Multiple stakeholders evaluated these materials; 37 Veterans Affairs staff, 6 older veterans (60+ years); the Veteran and Family Advisory Council |
| Jobling (2014) | Not stated. | Descriptive article. | N/A | Community Living Campaign (CLC), San Francisco | Peer tutors | Community setting | N/A |
| Leedahl, S.N., Brasher, M.S., Estus, E., Breck, B.M., Dennis, C.B., & Clark, S.C. (2019) | This study aimed to assess outcomes for older adults related to social connections and technology  use, and to understand attitudes toward aging and working with older adults. | Mixed | Multiple data collection methods were used: student logs of each  session, pre/post surveys, and reflection papers. | Three models were developed and adopted:  Individual appointments - 30-60 minute drop-in sessions in which older adult brought their own device and asked the student questions. There was an option to sign up for further sessions.  Matching programme – Each matched pair (older adult and student) met for at least 6 hours each semester. This was agreed by each pair.  Drop-in sessions – A group of students were available for 2-4 hour periods. | 1:1; intergenerational | Community setting (Lifelong learning institute) | Student mentors (n=28) and older adults (n=25) |
| LoBuono et al (2019) | The primary aim of this qualitative study is to describe  specific areas of technology  older adults  that are interested in using and why | Qualitative | Student observation logs | University of Rhode Island (URI)’s Engaging Generations: Cyber-Seniors Program | 1:1, Intergenerational | Senior centres throughout the state | 199 older adults; 27 student mentors |
| LoBuono, D.L., Leedahl, S.N., & Maiocco, E. (2020) | This study aimed to examine college student teaching in an intergenerational programme that helps older adults learn technology and described reasons for older adults’ continued participation in this program. | Qualitative | Observation logs | Students support older adults as part of the reverse-mentoring programme “The University of Rhode Island’s (URI) Engaging Generations: Cyber-Seniors (E-GEN)”.  The way in which students engaged in this teaching was part of the study’s findings. | 1:1; Intergenerational | Multiple | 27 student logs and 199 older adults’ logs were analysed. |
| Loi et al (2017) | The aims of this project were to investigate  (1) RACF staff current experience (such as their  confidence and frequency of use) using TTs before  these are introduced; (2) their perceptions about  engaging residents with TT (including how this  might help them care for the residents); and (3)  what methods of training would support them in  using TTs to engage residents. | Quantitative | Questionnaire | N/A | Semi-professional tutors | Residential aged care facilities (RACFs) | 62 participants representing different roles: registered nurse, enrolled nurse, personal care attendant, allied health, other (e.g. admin) |
| Segui et al (2019) | The aim of this paper is to evaluate the satisfaction of both junior and senior participants toward the intervention  and to explore its main drivers. | Mixed methods | Paper-based ad hoc surveys to assess participants’ satisfaction | Digital Partners, Catalonia | 1:1, Intergenerational | Training space within University of Vic | 42 junior users (aged 14-15 years) and 38 older adults (aged 65 years+). |
| Seo et al (2019) | To examine how a digital literacy class for older, low-income African-American adults has influence their use of digital technologies, verification of online information, as well as their perceptions of privacy and security online. | Mixed methods | Focus groups, participant observations, interviews, analysis of class documents and assignments | Name not stated | Professional tutor, Classroom setting | Senior community centre | 47 older, low-income African-American adults |
| Tomczyk et al (2020) | The goal of the study was to diagnose the needs of instructors working  in the area of the digital inclusion of persons who are excluded, at risk  of exclusion, marginalized, and discriminated against in terms of using  new technologies. | Qualitative | Interviews | Various | Various | Various | 8 educators of older adults, representing different types of institutions dealing with the digital divide: an activity centre, a public library, a nursing home, an NGO and University of the Third Age. |
| Xie et al (2012) | The aim is to focus on developing and assessing the effectiveness of a public library-based e-health literacy intervention designed specifically for older adults aged 60 and above. | Quantitative | Questionnaires | e-health literacy intervention:  NIHSeniorHealth.gov and MedlinePlus.gov | Professional tutor, Group classroom setting (no more than 7 trainees per class), using online training tools | Public libraries | 218 older adults, aged 60-89, 1 graduate student instructor |
